# Gated F-18 FDG PET for Assessment of Left Ventricular Volumes and Ejection Fraction Using QGS and 4D-MSPECT in Patients with Heart Failure: A Comparison with Cardiac MRI

**DOI:** 10.1371/journal.pone.0080227

**Published:** 2014-01-03

**Authors:** Yan Li, Li Wang, Shi-Hua Zhao, Zuo-Xiang He, Dao-Yu Wang, Feng Guo, Wei Fang, Min-Fu Yang

**Affiliations:** 1 Department of Nuclear Medicine, Cardiovascular Institute and Fu Wai Hospital, Peking Union Medical College and Chinese Academy of Medical Sciences, Beijing, China; 2 Department of Radiology, Cardiovascular Institute and Fu Wai Hospital, Peking Union Medical College and Chinese Academy of Medical Sciences, Beijing, China; 3 Department of Nuclear Medicine, Chaoyang Hospital, Capital Medical University, Beijing, China; Washington Hospital Center, United States of America

## Abstract

**Purpose:**

Ventricular function is a powerful predictor of survival in patients with heart failure (HF). However, studies characterizing gated F-18 FDG PET for the assessment of the cardiac function are rare. The aim of this study was to prospectively compare gated F-18 FDG PET and cardiac MRI for the assessment of ventricular volume and ejection fraction (EF) in patients with HF.

**Methods:**

Eighty-nine patients with diagnosed HF who underwent both gated F-18 FDG PET/CT and cardiac MRI within 3 days were included in the analysis. Left ventricular (LV) end-diastolic volume (EDV), end-systolic volume (ESV), and EF were obtained from gated F-18 FDG PET/CT using the Quantitative Gated SPECT (QGS) and 4D-MSPECT software.

**Results:**

LV EDV and LV ESV measured by QGS were significantly lower than those measured by cardiac MRI (both *P*<0.0001). In contrast, the corresponding values for LV EDV for 4D-MSPECT were comparable, and LV ESV was underestimated with borderline significance compared with cardiac MRI (*P* = 0.047). LV EF measured by QGS and cardiac MRI showed no significant differences, whereas the corresponding values for 4D-MSPECT were lower than for cardiac MRI (*P*<0.0001). The correlations of LV EDV, LV ESV, and LV EF between gated F-18 FDG PET/CT and cardiac MRI were excellent for both QGS (*r* = 0.92, 0.92, and 0.76, respectively) and 4D-MSPECT (*r* = 0.93, 0.94, and 0.75, respectively). However, Bland-Altman analysis revealed a significant systemic error, where LV EDV (−27.9±37.0 mL) and ESV (−18.6±33.8 mL) were underestimated by QGS.

**Conclusion:**

Despite the observation that gated F-18 FDG PET/CT were well correlated with cardiac MRI for assessing LV function, variation was observed between the two imaging modalities, and so these imaging techniques should not be used interchangeably.

## Introduction

Heart failure (HF) is a severe public health problem with escalating prevalence and high mortality [1]. Assessing left ventricular (LV) function and volume is of high clinical value in myocardial diseases [2], [3]. A variety of non-invasive techniques are available to assess LV volume and function, including 2-dimensional (2D) echocardiography, magnetic resonance imaging (MRI), radionuclide ventriculography, and gated single photon emission computed tomography myocardial perfusion imaging (SPECT-MPI) [4]–[10]. Gated SPECT, as a first-line diagnostic technique in coronary artery disease (CAD), has been widely used clinically to simultaneously assess myocardial perfusion and parameters of ventricular function. As such, several software packages for the quantification of ventricular function are available for clinical practice, including Quantitative Gated SPECT (QGS), 4D-MSPECT, and Emory Cardiac Toolbox (ECTB) [7], [11]–[12]. F-18 FDG PET/CT metabolic imaging with high resolution is an accurate, quantitative, and non-invasive imaging modality that is considered to be the gold standard for the identification of viable myocardium, and is used increasingly in clinical cardiology [13], [14]. Moreover, electrocardiographically (ECG) gated F-18 FDG PET/CT allows the combination of assessments of LV function and volume, as well as myocardial metabolism in a single PET/CT examination. However, studies on the evaluation of gated ^18^F-FDG PET for the assessment of cardiac function are rare [15]–[17]. Cardiac MRI is the preferred reference method for the determination of LV parameters because of its accuracy, reproducibility, and higher temporal and spatial resolution [18]–[20]. The aim of the study was therefore to investigate the consistency and correlation between gated F-18 FDG PET/CT using QGS and 4D-MSPECT software packages and cardiac MRI for the assessment of LV volume and function in HF patients.

## Materials and Methods

### Study population

One hundred and twenty-four consecutive hospitalized patients with known HF who underwent both F-18 FDG PET/CT and cardiac MRI at our hospital between October 2010 and November 2011 were included in the study. Myocardial Tc-99m sestamibi SPECT was also performed to assess viability or differentiate between etiologies (ischemic or non-ischemic) in combination with F-18 FDG PET/CT. The inclusion criteria were as follows: clinically diagnosed as HF (typical clinical symptoms: shortness of breath at rest or during exertion, fatigue, signs of fluid retention, and objective evidence of cardiac structural or functional abnormality at rest) [21]. Patients with hypertrophic cardiomyopathy, obstructive cardiomyopathy, valvular cardiomyopathy, right-sided heart failure, arrhythmia, and aneurysm documented by angiography or echocardiography were excluded. Of the 124 patients originally identified, 35 were excluded for 21 with LV aneurysm, 5 with hypertrophic cardiomyopathy, 2 with valvular cardiomyopathy, 4 with arrhythmia, and 3 with poor uptake of F-18 FDG due to insulin resistance. Finally, 89 patients (69 male and 20 female; mean age 54.7±13.1 years) were enrolled, consisting of 47 patients with a history of CAD, and 42 patients with diagnosed DCM (normal coronary arteries on coronary angiography and/or coronary computed tomography angiography). Diabetes mellitus had been diagnosed in 45 patients (50.6%). The mean fasting blood glucose levels were 7.1±2.1 mmol/L. Cardiac MRI was performed within 2–7 days of F-18 FDG PET/CT. The Ethics Committee of Fu Wai Hospital approved the study, and written informed consent was obtained from all patients.

### Tc-99m MIBI SPECT/F-18 FDG PET/CT imaging

Myocardial Tc-99m MIBI SPECT acquisition was performed using a dual-head gamma camera (e.cam, Siemens Medical Solution, Inc.), and images were acquired using standard protocols [22]. Myocardial F-18 FDG PET/CT imaging was performed within 2 days using SPECT. After a minimum of 12 hours overnight fast, all patients were given a 25–50 g oral glucose load before the injection of F-18 FDG (3 MBq/kg) [23], [24]. Images were acquired 1–2 h later using PET/CT (Truepoint Biography 64, Siemens Medical Solutions, Knoxville, TN). The acquisition time was 10 minutes for emission, and the gated acquisition mode was 8 frames per cardiac cycle. Images were reconstructed using attenuation weighted-OSEM iterative algorithm (4 iterations, 8 subsets).

Gated F-18 FDG PET/CT images were analyzed for functional parameters (LV EDV, LV ESV and LV EF) using two different software packages: QGS (version 3.1, Cedars-Sinai Medical Center, Los Angeles, CA, USA) and 4D-MSPECT (version 3.0, University of Michigan Medical Center, Ann Arbor, MI, USA) on a Siemens e.soft workstation. The reconstruction and data analyses were performed by an observer who was unaware of the results of cardiac MRI. To avoid bias in the data interpretation, QGS and 4D-MSPECT analyses were carried out at different times. Automatic processing was used for all algorisms, with the option of manual correction in case of obvious misalignment of the cardiac contour. LV EDV and LV ESV values were reported in milliliters (mL), and LV EF values were reported as percentages (%).

Myocardial F-18 FDG PET images were read in combination with resting SPECT images for the assessment of myocardial viability [24]. The extent of viable myocardium (mismatch, %) and scar (scar, %), and the perfusion defect (TPD) was expressed as the percent of LV area. In addition, the ^18^F-FDG-uptake ratio (SUV_M/B_) was measured to assess the contrast between the myocardium and blood-pool [25]. An experienced nuclear physician using Syngo TrueD software processed the images. A circular region of interest (ROI) was manually drawn on the view, and adjusted to ensure that the whole left ventricle was included in all 3 dimensions. The mean SUV of LV myocardium (SUV_myo_) was then obtained automatically. The ROI of the cardiac blood pool was drawn in the localization of mitral valve in 3-dimensions and the background and the mean SUV of blood pool (SUV_blood_) were obtained. The ratio of SUV_myo_ to SUV_blood_ was then calculated as SUV_M/B_ [25].

### Cardiac MRI

Cardiac MRI is an imaging modality that does not rely on geometric assumptions or calculations based on incomplete sampling of the cardiac volumes to assess cardiac function. It is highly reproducible, and is considered superior to other imaging techniques because of its ability to produce high-resolution measurements of the anatomy and function of the left ventricle [26]. The imaging protocol used in the study was previously described [22], [27], [28]. Briefly, cardiac MRI data were acquired on a 1.5-T system (Magnetom Avanto, Siemens), and the breath-hold technique true fast imaging with steady-state procession cine cardiac MRI was used to obtain images with a superior signal-to-noise ratio [27]. Continuous slices encompassing the entire left ventricle from apex to base were obtained during breath-hold using the following parameters: slice thickness, 8 mm; matrix, 256×256; 25 phases per cardiac cycle; and a field of view of 350–400 mm. Each slice was acquired in a separate breath-hold cycle after expiration.

**Figure 1:**
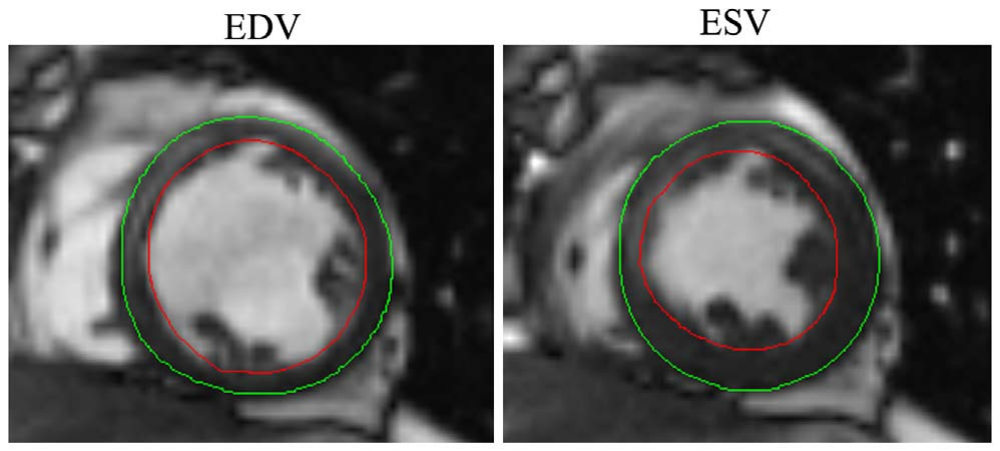
The delineations for the modalities are shown on short axis slices of MRI (end-diastolic and end-systolic).

One physician unaware of the clinical and gated PET/CT data analyzed the images using MASS software (version 5.0; Medis Medical Imaging Systems, Leiden, The Netherlands). The first frame of the stack in each slice was selected as the end-diastolic image, and the end-systolic image was selected as the image with the smallest ventricular volume. Endocardial and epicardial borders were outlined manually on both the end-diastolic and end-systolic frames (Fig. 1). The trabeculation and papillary muscles were segmented as part of the myocardium, and were excluded from LV volumes. LVEDV and LVESV were computed in milliliters using the modified Simpson's rule [20], and the LVEF was expressed as a percentage (%).

### Statistical analysis

Statistical analysis was performed using SPSS 19.0 software (SPSS Inc., Chicago, IL). Data were expressed as mean ± SD for continuous variables, and percentages or numbers for categorical variables. The mean values of LVEDV, LVESV, and LVEF were assessed for significance using a *t* test for paired samples with the application of correlation and regression analysis. Bland-Altman analysis was used to evaluate the degree of agreement and to assess any systematic trends in differences [29]. The correlation coefficient (*r*) and Bland-Altman limit (BAL) were calculated. To test the processing variability of F-18 FDG PET/CT and cardiac MRI, all datasets were processed again by the same and another experienced observer to evaluate the intra- and inter-observer reproducibility of the LV parameters. Significant differences were defined as *P* values<0.05.

## Results

### Patient characteristics

The characteristics of the study population are summarized in Table 1.

**Table 1:** Patient characteristics.

Parameters	Values
Male (*n*, %)	69 (77.5)
Age (y, mean ± SD)	54.7±13.1
New York Heart Association	
II (*n*, %)	27 (30.3)
III (*n*, %)	44 (49.4)
IV (*n*, %)	18 (20.2)
Disease type	
Ischemia (*n*, %)	47 (52.8)
Non-ischemia (*n*, %)	42 (47.2)
Diabetes mellitus (*n*, %)	45 (50.6)
Fasting glucose (mmol/L, mean ± SD)	7.1±2.1
Hypertension (*n*, %)	56 (62.9)
Hyperlipidemia (*n*, %)	37 (41.6)
SUV _T/B_ (mean ± SD)	5.8±3.8
SRS (mean ± SD)	12.2±10.4
TPD (mean ± SD)	15.8±13.9
Mismatch (%, mean ± SD)	6.8±7.7
Scar (%, mean ± SD)	9.1±10.2
LV EDV (ml, mean ± SD)	220.3±90.7
LV ESV (ml, mean ± SD)	158.9±85.8
LV EF (%, mean ± SD)	31.3±12.2

*SUV_T/B_* = the ratio of the standardized uptake value of F-18 FDG between the contrast and the myocardium; *SRS* = summed rest score; *TPD* = total perfusion deficit; *LVEDV* = left ventricular end-diastolic volume; *LVESV* = left ventricular end-systolic volume; *LVEF* = left ventricular ejection fraction.

### LV volumes and EF by cardiac MRI and gated F-18 FDG PET/CT

**Figure 2:**
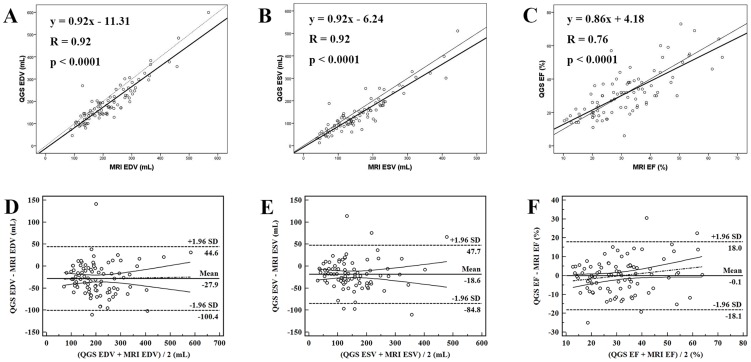
Correlation analyses of LVEDV (A), LVESV (B), and LVEF (C) estimated using QGS from gated F-18 FDG PET/CT and cardiac MRI in 89 HF patients. Bland-Altman plots of comparisons of QGS versus cardiac MRI for the measurements of LVEDV (D), LVESV (E), and LVEF (F).

**Figure 3:**
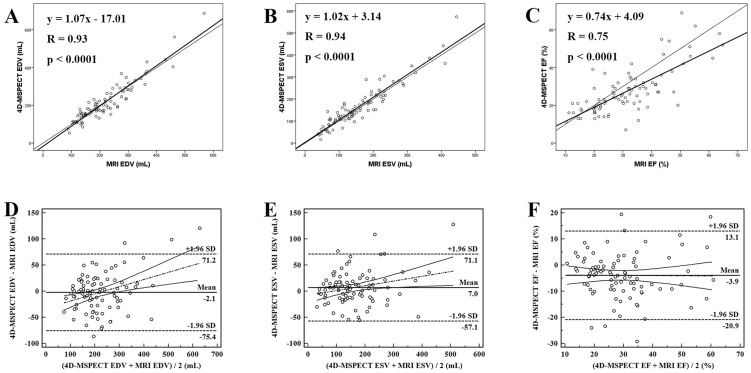
Correlation analyses of LVEDV (A), LVESV (B), and LVEF (C) estimated with 4D-MSPECT from gated F-18 FDG PET/CT and cardiac MRI in 89 HF patients. Bland-Altman plots of comparisons for 4D-MSPECT versus cardiac MRI for the measurements of LVEDV (D), LVESV (E), and LVEF (F).

The quantitative measurements of LV volumes and EF are shown in Table 2. LV EDV measured by cardiac MRI ranged from 88 to 568 mL (mean 220.3±90.7 mL). The corresponding values for QGS of gated F-18 FDG PET/CT (range 46 to 599 mL; mean 192.3±91.4 mL; *P*<0.0001) were significantly lower than the comparable cardiac MRI values. In contrast, the corresponding values for 4D-MSPECT of gated F-18 FDG PET/CT (range 53 to 688 mL; mean 218.1±103.6 mL; *P* = 0.595) were not significantly different from the cardiac MRI values. The correlation between the EDV of gated F-18 FDG PET/CT with the EDV of MRI was very high for QGS (*r* = 0.92, Fig. 2A) and 4D-MSPECT (*r* = 0.93, Fig. 3A).

**Table 2:** Left ventricular parameters assessed by gated F-18 FDG PET and cardiac MRI.

Parameter	Gated F-18 FDG PET		Cardiac MRI
	QGS	4D-MSPECT	
LVEDV (mL)	192.3±91.4* (46–599)	218.1±103.6 (53–688)	220.3±90.7 (88–568)
LVESV (mL)	140.3±85.8* (13–511)	165.8±93.8* (16–573)	158.9±85.8 (38–445)
LVEF (%)	31.2±13.9 (6–73)	27.4±12.2* (7–69)	31.3±12.2 (10–64)

*P*<0.05 vs. cardiac MRI (paired *t*-test).

Data are mean ± SD, with the range in parentheses.

*LVEDV* = left ventricular end-diastolic volume; *LVESV* = left ventricular end-systolic volume; *LVEF* = left ventricular ejection fraction.

Bland-Altman analysis revealed that LV EDV was underestimated by QGS (−27.9±37.0 mL; bias significantly different from 0, *P* = 0.004) with Bland-Altman limits of −100.4 mL to 44.6 mL. No significant trend was found for the estimation of LV EDV by QGS of gated F-18 FDG PET/CT (*P* = 0.86) (Fig. 2D). In contrast, no systemic error was revealed by Bland-Altman analysis (*P* = 0.60) for 4D-MSPECT, with limits of −75.4 mL to 71.2 mL. Compared with cardiac MRI, 4D-MSPECT of gated F-18 FDG PET/CT showed a trend towards higher LV EDV with increasing magnitude of LV EDV, as noted on the Bland-Altman plot (y = 0.14x−32.26, *P* = 0.0007) (Fig. 3D).

LV ESV measured by cardiac MRI ranged from 38 to 445 mL (mean 158.9±85.8 mL). The corresponding values for QGS of gated F-18 FDG PET/CT (range 13 to 511 mL; mean 140.3±85.8 mL; *P*<0.0001) were significantly lower than those of cardiac MRI. Similarly, the values for 4D-MSPECT of gated F-18 FDG PET/CT (range 16 to 573 mL; mean 165.8±93.8 mL; *P* = 0.047) were also lower than those of cardiac MRI. The correlation between the ESV of gated F-18 FDG PET/CT and the ESV of MRI was very high for QGS (*r* = 0.92, Fig. 2B) and 4D-MSPECT (*r* = 0.94, Fig. 3B).

Bland-Altman analysis revealed that LV ESV was underestimated by QGS (−18.6±33.8 mL; bias significantly different from 0, *P*<0.0001), with Bland-Altman limits of −84.8 mL to 47.7 mL. No significant trend was identified for the estimation of LV ESV by QGS of gated F-18 FDG PET/CT (*P* = 1.00) (Fig. 2E). In contrast, the LV ESV for 4D-MSPECT was overestimated (7.0±32.7 mL; bias significantly different from 0, *P* = 0.047), with Bland-Altman limits of −57.1 mL to 71.1 mL. Compared with cardiac MRI, the 4D-MSPECT of gated F-18 FDG PET/CT showed a trend towards higher LV ESV with increasing magnitude of LV ESV, as revealed on the Bland-Altman plot (y = 0.09x−7.83, *P* = 0.03) (Fig. 3E).

The LV EF measured by cardiac MRI ranged from 10–64% (mean 31.3±12.2%). The corresponding values for QGS of gated F-18 FDG PET/CT (range 6 to 73%; mean, 31.2±13.9%; *P* = 0.95) showed no significant difference compared with cardiac MRI. In contrast, the values for 4D-MSPECT of gated F-18 FDG PET/CT (range 7 to 69%; mean 27.4±12.2%; *P*<0.0001) were significantly lower than for cardiac MRI. The correlation between the LV EF of gated F-18 FDG PET/CT and the EF of MRI was high for both QGS (*r* = 0.76, Fig. 2C) and 4D-MSPECT (*r* = 0.75, Fig. 3C).

No systemic error for the estimation of LVEF by QGS was revealed (Bland-Altman limits, −18.1% to 18.0%) compared with cardiac MRI (Fig. 2F). In contrast, Bland-Altman analysis revealed that LVEF was underestimated by 4D-MSPECT (−3.9±8.7%; bias significantly different from 0, *P*<0.0001) with Bland-Altman limits of −20.9% to 13.1% (Fig. 3F). No significant trends were detected for the estimation of LV EF by QGS and 4D-MSPECT.

### Variations in LV volumes by cardiac MRI and gated F-18 FDG PET/CT

The relationship between the gated F-18 FDG PET/CT parameters and the difference between LV volumes assessed by cardiac MRI and gated F-18 FDG PET/CT were investigated. The underestimation of LV EDV or LV ESV by gated F-18 FDG PET/CT compared with MRI was not associated with the contrast of FDG uptake (SUV _M/B_) between myocardium and blood-pool, the percentage of perfusion defects, viable myocardium, or scar.

### Reproducibility

The reproducibility of LV parameters measured by gated F-18 FDG PET/CT and cardiac MRI were presented in Table 3. Both intra- and inter-observer comparisons of repeated measurements of LV parameters show significant correlations in both gated F-18 FDG PET/CT and cardiac MRI.

**Table 3:** Reproducibility of gated F-18 FDG PET and cardiac MRI parameters.

Difference	LVEDV (mL)			LVESV (mL)			LVEF (%)		
	QGS	4D-MSPECT	MRI	QGS	4D-MSPECT	MRI	QGS	4D-MSPECT	MRI
Intra-observer	0.96	0.93	0.92	0.98	0.94	0.93	0.91	0.95	0.90
Inter-observer	0.97	0.95	0.94	0.99	0.93	0.92	0.93	0.93	0.91

Significant correlations (*r*) between the repeated measurements revealed excellent reproducibility (all *P*<0.05).

## Discussion

Our study showed high correlations between gated F-FDG PET/CT and cardiac MRI for measurements of LV EDV, ESV, and EF using QGS and 4D-MSPECT. A comparison between QGS and cardiac MRI revealed excellent correlations, but a significant underestimation of LVEDV and ESV, but not LV EF. A comparison between 4D-MSPECT and cardiac MRI showed slightly better correlations than QGS (EDV: *r* = 0.93 vs. 0.92; ESV *r* = 0.94 vs. 0.92), no underestimation of LV EDV and ESV, but borderline significance for overestimating LV ESV (*P* = 0.047) and a significant underestimation of LV EF was observed.

The reasons for variability between the LV parameters for gated F-18 FDG PET/CT and cardiac MRI were investigated. However, we did not find any relationship between the differences in LV volumes and the extent or severity of FDG defects, in contrast FDG uptake between the myocardium and blood-pool or fasting glucose levels. The influence of these factors can therefore be ruled out, suggesting that the variability may be attributable to differences in the method of imaging and the automatic algorithms.

There are several possible reasons that could explain the differences in the measurements of the LV volumetric parameters between the two techniques. First, there is a much lower temporal resolution of gated FDG PET (8 gates per cardiac cycle) compared with MRI (25 gates per cardiac cycle). This is consistent with the findings of Kumita et al. [30], who evaluated data from the same study using 8 gates per cycle, and then 16 gates per cycle. This resulted in significant variability between the results of each analysis. The lower temporal resolution of gated F-18 FDG PET/CT (8 gates per cardiac cycle) compared with MRI (25 gates per cardiac cycle) may cause blurring of the end-systolic and end-diastolic phases, resulting in variability in volumetric measurements. Second, there was a difference in the positioning of the myocardial cavity. PET/CT excluded the outflow tract tissue for the low counts in this area, whereas MRI allows its inclusion, which may cause a larger EDV in cardiac MRI. Third, the motion or respiratory motion of the patient during PET/CT could influence the measurement of LV volumes, even though although instructions were given to the patient before data acquisition. Fourth, we did not find any significant relationship between FDG uptake (by contrast and the myocardium) or myocardial FDG defects and volumetric variability (possibly due to the limited study population). However, the fact that “hibernating myocardium” frequently shows perfusion deficits while glucose metabolism is preserved would suggest that gated FDG-PET has advantages over myocardial perfusion imaging, where delineation can be complicated by perfusion deficits [31]. These factors may have a greater impact in a larger study population. Moreover, the chosen reference method, cardiac MRI, is not the perfect gold standard for measuring LV volumes and EF, even though it is accepted as the current best available method. MRI provides nearly, but not truly, three-dimensional data. The two-dimensional short-axis slices of cardiac MRI are acquired separately, not simultaneously, because the position of the heart is not exactly reproducible in different both-hold cycles and the valve plane motion is difficult to assess from short-axis images. In addition, the LV volumes calculated by cardiac MRI relied on manually delineated borders of the myocardium, which increases the risk of mis-delineation of the myocardial contours.

Our previous research demonstrated that SUV_M/B_ was one of the factors associated with the overestimation of PET/CT for assessing left ventricular dyssynchrony [25]. In this study, however, we did not find any association of SUV_M/B_ with the underestimation of LV volumes by gated F-18 FDG PET/CT, probably due to the small study population. It is well known that the uptake of F-18 FDG by the myocardium mainly depends on substrate availability and hormonal status. Poor uptake of F-18 FDG can lead to mis-delineation by gated PET. However, there has been little previous evidence for a significant influence of the glucose and insulin levels of patients. This is consistent with our observation that no significant impact was caused by the large number of patients with diabetes mellitus and high fasting blood glucose levels in our study population [15]–[17].

Measurements of LV EDV, ESV, and EF are clinically relevant, particularly in patients with CAD before CABG. The LV volumetric parameters were demonstrated to be predictors of long-term survival in patients with ischemic cardiomyopathy [32], [33]. Gated SPECT has added greatly to this clinical value. F-18 FDG PET/CT, with higher resolution than SPECT, can minimize the uncertainty of the exact position of the myocardial wall, and may therefore better estimate LVEF, LVEDV, and LVESV than gated SPECT [34]. Different tracers for PET have been developed, including N-13 NH_3_ and Rb-82, and comparisons of these modalities for the assessment of LV function have been investigated [35], [36]. Comparisons of measures of left ventricular function from electrocardiographically gated Rb-82 PET with contrast-enhanced CT ventriculography demonstrated that global LV function could be measured reproducibly from gated Rb-82 PET using different available software products, and that these analyses resulted in an underestimation of EF [36]. Combing gated information with metabolism match and mismatch patterns on gated F-18 FDG PET/CT may be more beneficial for data interpretation. Gated F-18 FDG PET allows a complete LV evaluation with a single technique to evaluate major clinical parameters including metabolism, flow reserve, and LV function, with no additional scanning time or tracer injection.

Comparisons of measurements between PET and MRI have been reported [15]–[17], [28]. Schaefer et al. found high correlations between F-18 FDG PET and cardiac MRI for EDV, ESV, and LVEF (*r* = 0.96, 0.97, and 0.95, respectively) using QGS for gated PET quantification [15]. They also observed a good correlation between cardiac MRI and gated F-18 FDG PET/CT using QGS and 4D-MSPECT in 42 patients with severe CAD [16]. The correlations identified in previous studies are somewhat higher than observed in our study, which may be a result of the different study populations. We previously demonstrated an excellent correlation of LV function between gated PET and cardiac MRI in DCM patients (LVEDV, *r* = 0.948; LVESV, *r* = 0.939; LVEF, *r* = 0.685; all *P*<0.05) [28]. The underlying reasons for the variability between comparisons of different measurements remain unclear.

Our study has several limitations. First, the manual method of outlining the contours of the myocardium on a cardiac MRI may result in bias. Second, a higher number of frames per cycle may have provided a more accurate assessment.

## Conclusion

Despite an excellent agreement between cardiac MRI and gated F-18 FDG PET/CT in the assessment of left ventricular function, there was variation between the two imaging modalities. These two imaging techniques should therefore not be used interchangeably.
